# Mechanical thrombectomy for emergent large vessel occlusion: a critical appraisal of recent randomized controlled clinical trials

**DOI:** 10.1002/brb3.418

**Published:** 2016-01-07

**Authors:** Georgios Tsivgoulis, Apostolos Safouris, Aristeidis H. Katsanos, Adam S. Arthur, Andrei V. Alexandrov

**Affiliations:** ^1^ Department of NeurologyUniversity of Tennessee Health Science CenterMemphisTennessee; ^2^ Second Department of Neurology“Attikon University Hospital”School of MedicineUniversity of AthensAthensGreece; ^3^ International Clinical Research CenterSt. Anne's University Hospital in BrnoBrnoCzech Republic; ^4^ Stroke UnitDepartment of NeurologyBrugmann University HospitalBrusselsBelgium; ^5^ Stroke UnitMetropolitan HospitalAthensGreece; ^6^ Department of NeurosurgerySemmes‐Murphey Neurologic and Spine InstituteUniversity of Tennessee Health Science CenterMemphisTennessee

**Keywords:** Endovascular treatment, intravenous thrombolysis, ischemic stroke, recanalization, thrombectomy

## Abstract

**Background and Purpose:**

After numerous attempts to prove efficacy for endovascular treatment of ischemic stroke, a series of recent randomized controlled clinical trials (RCTs) established fast mechanical thrombectomy (MT) as a safe and effective novel treatment for emergent large vessel occlusion (ELVO) in the anterior cerebral circulation.

**Methods:**

We reviewed five recent RCTs that evaluated the safety and efficacy of MT in ELVO patients and captured available information on recanalization/reperfusion, symptomatic intracranial hemorrhage (sICH), clinical outcome, and mortality. MT was performed with stent retrievers, aspiration techniques, or a combination of these endovascular approaches. We applied meta‐analytical methodology to evaluate the pooled effect of MT on recanalization/reperfusion, sICH, functional independence (modified Rankin scale score of 0–2) and 3‐month mortality rates in comparison to best medical therapy (BMT).

**Results:**

MT was associated with increased likelihood of complete recanalization/reperfusion (RR: 2.22; 95%CI: 1.89–2.62; *P* < 0.00001) and 3‐month functional independence (RR: 1.72; 95%CI: 1.48–1.99; *P* < 0.00001) without any heterogeneity across trials (*I* ^2^ = 0%). The absolute benefit increase in MT for complete recanalization/reperfusion and functional independence was 44 (NNT = 2) and 16 (NNT = 6), respectively. MT was not associated with increased risk of 3‐month mortality (15% with MT vs. 19% with BMT) and sICH (4.6% with MT vs. 4.3% with BMT), while small heterogeneity was detected across the included trials (*I* ^2^ < 25%).

**Conclusions:**

MT is a safe and highly effective treatment for patients with ELVO in the anterior circulation. For every six ELVO patients treated with MT three more will achieve complete recanalization at 24 h following symptom onset and one more will be functionally independent at 3 months in comparison to BMT.

## Introduction

For almost 20 years intravenous thrombolysis using recombined tissue plasminogen activator (IVTPA) has been the only FDA‐approved therapy for acute ischemic stroke (AIS). IVTPA turns inactive plasminogen into active plasmin, a proteolytic enzyme that breaks fibrin cross‐links, leading eventually to fibrin clot dissolution (Barreto 2011) and, hopefully, to recanalization of an occluded brain artery. When outcome at 3 months poststroke is assessed using the modified Rankin scale of global disability, IVTPA given up to 3 h after symptom onset produces a beneficial shift in outcome in one of the three patients treated, whereas one in 33 patients is harmed by this therapy. Half of this benefit has been reported for patients treated in the 3–4.5 h window with practically the same risk for harm (Saver et al. 2009). IVTPA after 4.5 h is associated with increased mortality (Lees et al. 2010). Residual blood flow in the affected artery as measured using transcranial Doppler (TCD) is a major determinant of both initial stroke severity and favorable response to IVTPA (Demchuk et al. 2001). Rapid and complete artery recanalization has been shown to lead to better outcomes (Alexandrov et al. 2001). A meta‐analysis has reported a strongly positive correlation between brain artery recanalization, spontaneous or posttreatment, and good clinical outcome with an odds ratio of 4.4% and a 76% reduction in mortality (Rha and Saver 2007). However, in the aforementioned study, IVTPA led to vessel recanalization in less than half of the patients treated, whereas one in four patients achieved spontaneous recanalization without IVTPA. Moreover, arterial reocclusion is not infrequent, reported in up to a third of patients treated with IVTPA after initial recanalization (Alexandrov and Grotta 2002). IVTPA has not been shown to be effective after 4.5 h, limiting treatment to a small subgroup of patients (Weczrhsler 2011). The population of treatable patients is further truncated by a long list of contraindications, some of which are relative but still restrain treatment use (Tsivgoulis et al. 2015). Finally, large clots located in the terminal internal carotid artery or proximal middle cerebral artery have little chance of recanalization with IVTPA. More specifically, thrombi longer than 8 mm measured in thin‐slice nonenhanced brain CT scans were found to be almost impossible (<1%) to dissolve with IVTPA in a recent single‐center study (Riedel et al. 2011). In short, the benefit for stroke patients receiving IVTPA is hampered by a short window of opportunity, many exclusion criteria, and most importantly, by only partial efficacy in achieving complete and lasting artery recanalization especially in cases with high clot burden.

Endovascular stroke therapies have been evolving to respond to these therapeutic challenges. Several different endovascular approaches have been introduced that have achieved high recanalization rates and improved clinical benefits in the treatment of myocardial infarction (Saver 2013). First of all, intra‐arterial administration of tissue plasminogen activator has been tested after reduced IVTPA dose (bridging therapy) or directly without IVTPA. The theoretical advantages of delivering a fibrinolytic agent directly to the clot through a microcatheter are reduced systemic exposure reducing extracranial bleeding risk and increased concentration in the occluded vessel where the mechanical disruption using the catheter wire may increase the odds of recanalization. Despite a higher rate of complete recanalization, no clinical trial has shown a statistically significant improvement in clinical outcomes with this approach compared to standard treatment. It is likely that any benefit from improved recanalization rates was counterbalanced by the longer time needed for endovascular treatment (Hennerici et al. 2013). The same held true for trials testing thrombus aspiration catheters and first‐generation clot retrieval devices that provided preliminary evidence that an effective recanalization of ELVO can be translated into improved clinical outcome only when achieved early enough to prevent irreversible ischemia (Broderick, 2013).

The development of stent retrieval thrombectomy devices for ELVO was a major breakthrough that brought the advantage of rapid flow restoration after stent expansion without necessitating subsequent aggressive antiplatelet therapy as the stent is removed from the occluded vessel at the end of the procedure. The superiority of these second‐generation clot retrieval devices compared to the first generation has been shown in two head‐to‐head randomized controlled clinical trials (RCTs): the Solitaire With the Intention For Thrombectomy (SWIFT) trial (Saver et al. 2012) and the Thrombectomy Revascularization of Large Vessel Occlusions in Acute Ischemic Stroke (TREVO 2) trial (Nogueira et al. 2012). Both Solitaire and Trevo stent retrievers, respectively, were associated with higher recanalization and greater functional independence rates with the additional benefit of the Solitaire on mortality and symptomatic intracranial hemorrhage (sICH).

Still, we had to wait until late 2014 for the first positive RCTs testing mechanical thrombectomy (MT) in addition to and in comparison to best medical therapy (BMT). One obvious reason for the lack of benefit with earlier studies was the use of older generation thrombectomy devices. Stent retrievers have been proposed over older coil retrievers since 2013 by the American Heart Association/American Stroke Association (AHA/ASA) guidelines for the early management of patients with AIS (Jauch et al. 2013). A second reason was the prolonged onset‐to‐recanalization time in earlier studies. In the Interventional Management of Stroke (IMS) III trial (Broderick et al. 2013), a delay of 130 min was noted between IVTPA bolus and endovascular recanalization and it had already been shown from the precedent IMS I and II trials that a 30‐min treatment delay led to a 10% reduction in the probability of functional independence (IMS Study Investigators, 2004; The IMS II Trial Investigators, 2007). This association was also confirmed for the IMS III trial; in a preplanned analysis using predefined confounding variables. More specifically, every 30‐min delay in angiographic reperfusion resulted in a statistically significant 12% relative risk reduction for good clinical outcome after multivariate adjustment (Khatri et al. 2014). Others have reported that for every 5‐min delay in reperfusion after thrombectomy, one of the 100 patients has a worse disability outcome (Sheth et al. 2015). Third, patients were included who did not have an emergent large vessel occlusion; in fact, IMS III suggested a benefit for the subgroup of patients with large vessel occlusion on preintervention CT angiography. Finally, there was limited use of more advanced neuroimaging techniques for selecting patients with good collateral circulation and larger penumbra that would most likely benefit from treatment.

The present review attempts to critically appraise the five recent RCTs that evaluated the safety and efficacy of MT for patients with ELVO. We captured available information on recanalization/reperfusion, sICH, clinical outcome, and mortality. We applied meta‐analytical methodology to evaluate the pooled effect of MT on recanalization/reperfusion, sICH, 3‐month functional independence (modified Rankin scale score of 0–2), and 3‐month mortality rates in comparison to BMT.

### Statistical analyses

For each study, the numbers of events in patients with MT and BMT were identified and a risk ratio (RR) was calculated. For studies with a zero cell, we used a continuity correction of 0.5, as appropriate. In cases of ≥2 zero cells, the assumption of continuity correction was not used and the corresponding point estimates were designated as not estimable. The overall RR for all pooled studies was computed using the random effects method (DerSimonian and Laird). Heterogeneity between studies was assessed by the Cochran *Q* and *I* ^2^ statistic as described previously (Khan et al. 2014). Heterogeneity was considered as statistically significant when the *P* value derived from Cochran *Q* was <0.1. For the qualitative interpretation of heterogeneity, *I* ^2^ values of at least 50% are usually considered to represent substantial heterogeneity, whereas values of at least 75% indicate considerable heterogeneity as described previously (Khan et al. 2014).

## Results

### Overview of recent published RCTs for MT with stent retrievers

The first clinical trial that reported better clinical outcomes with thrombectomy for ELVO was the Multicenter Randomized Clinical Trial of Endovascular Treatment for Acute Ischemic Stroke in the Netherlands (MR CLEAN) published in December 2014 (Berkhemer et al. 2015). Three months after the procedure, one additional patient reached functional independence (modified Rankin scale 0–2) for every seven patients with thrombectomy. After disclosure of these encouraging results in October 2014, three other trials were prematurely halted and interim analyses also showed statistically significant clinical benefit: the Endovascular Treatment for Small Core and Anterior Circulation Proximal Occlusion with Emphasis on Minimizing CT to Recanalization Times (ESCAPE) trial (Goyal et al. 2015), the Extending the Time for Thrombolysis in Emergency Neurological Deficits – Intra‐Arterial (EXTEND‐IA) trial (Campbell et al. 2015), and the Solitaire with the Intention for Thrombectomy as Primary Endovascular Treatment (SWIFT PRIME) trial (Saver et al. 2015a,b). The results of a fifth positive trial have been most recently presented, the Randomized Trial of Revascularization with Solitaire FR Device versus Best Medical Therapy in the Treatment of Acute Stroke Due to Anterior Circulation Large Vessel Occlusion Presenting within Eight Hours of Symptom Onset (REVASCAT) (Jovin et al. 2015).

An overview of the inclusion criteria for these five RCTs is presented in Table 1. The time windows in these trials was short ranging between 6 h (MR CLEAN, SWIFT PRIME and EXTEND‐IA) and 12 h (ESCAPE), while the time window in REVASCAT was 8 h. Only patients with ELVO in the anterior circulation were included in all trials, while M2 middle cerebral artery occlusions were excluded from ESCAPE, SWIFT PRIME, and REVASCAT. The median National Institutes of Health Stroke Scale (NIHSS) score at randomization was around 17 points in both treatment groups (Table 2) indicating a moderate‐to‐severe neurological deficit that corresponds to an AIS due to an underlying large artery occlusion.

**Table 1:** Inclusion criteria across different randomized controlled clinical trials (RCTs)

RCT	Time window (h)	Affected arteries	Lowest NIHSS	Age limit	Neuroimaging	Lowest ASPECTS
MR CLEAN	<6	TICA, M1, M2, A1, A2	2	–	CT/CTA	–
ESCAPE	<12	TICA, M1	6	–	CT/CTA/CTA Multiphase (for collaterals)	6
EXTEND‐IA	<6	TICA, M1, M2	–	–	CT/CTA/CTP (for mismatch)	–
SWIFT PRIME	<6	TICA, M1, M2	8	<80	CT/CTA/MRA/MRP/CTP (for infarct core)	6
REVASCAT	<8	TICA, M1	6	<80	CT/CTA (MRA/DSA)	7 (CT) 6 (DWI)

TICA, terminal internal carotid artery; M1 and M2, branches of the middle cerebral artery; A1 and A2, branches of the anterior cerebral artery; NIHSS, National Institutes of Health Stroke Scale; ASPECTS, Alberta Stroke Program Early CT score; DWI, diffusion weighted imaging.

**Table 2:** Median National Institute of Health Stroke Severity Scores with corresponding interquartile ranges in both treatment arms of randomized controlled clinical trials (RCTs)

RCT	MT	BMT
MR CLEAN	17 (14–21)	18 (14–22)
ESCAPE	16 (13–20)	17 (12–20)
EXTEND‐IA	17 (13–20)	13 (9–19)
SWIFT PRIME	17 (13–20)	17 (13–19)
REVASCAT	17 (14–20)	17 (12–19)

MT, mechanical thrombectomy; BMT, best medical therapy.

Stent retrievers were used in 100% of patients randomized in the MT arm in EXTEND‐IA, SWIFT PRIME, and REVASCAT, while the vast majority of patients in the MT arms of MR CLEAN (82%) and ESCAPE (86%) were also treated with stent retrievers. Intravenous thrombolysis was coadministered in every patient enrolled in the EXTEND‐IA and SWIFT PRIME trials and in the majority of patients in both treatment arms in the remaining three trials (87% and 91% in the MT and BMT arm in MR CLEAN, respectively; 73% and 79% in the MT and BMT arm in ESCAPE, respectively; 68% and 78% in the MT and BMT arms in REVASCAT, respectively). The median elapsed time from symptom onset to groin puncture was <6 h in all trials ranging between 210 min in EXTEND‐IA (interquartile range 166–251) and 269 min in REVASCAT (interquartile range 201–340). The median time from groin puncture to revascularization was <1 h in the trials that provided relevant information (30 min in ESCAPE for first reperfusion, 43 min in EXTEND‐IA for complete reperfusion, and 59 min in REVASCAT for revascularization), indicating that MT with stent retrievers is an intervention that can rapidly restore blood flow in patients with ELVO.

**Figure 1:**
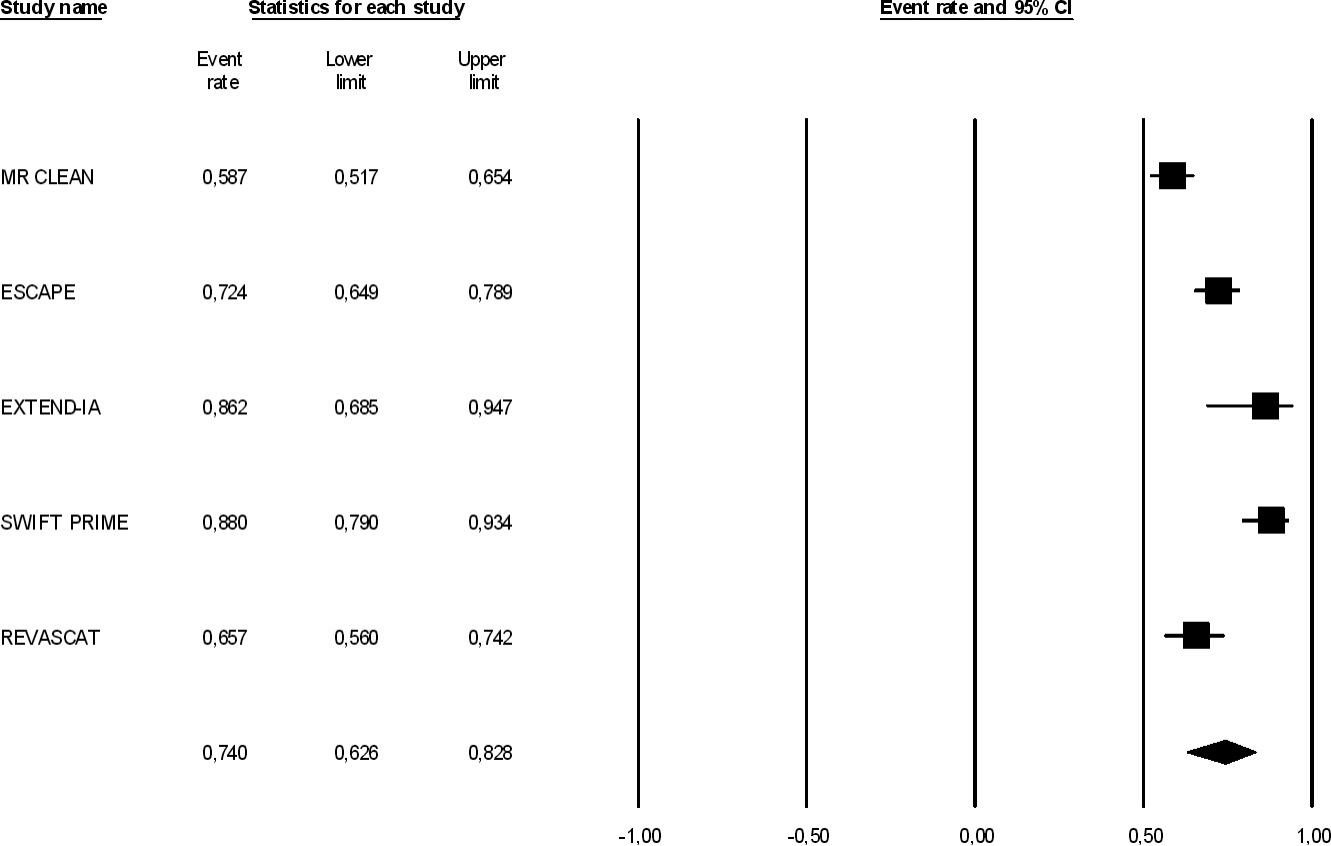
Rates of complete recanalization in patients with emergent large vessel occlusion (ELVO) treated with mechanical thrombectomy (MT) across different RCTs. Complete recanalization was defined as a modified Thrombolysis in Cerebral Infarction (TICI) score IIb or III at the end of the intervention.

**Figure 2:**
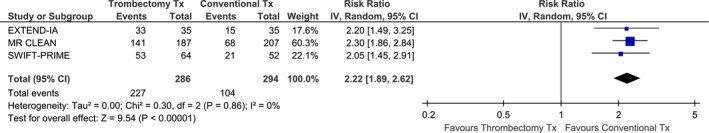
Association of mechanical thrombectomy (vs. best medical therapy) with the likelihood of complete recanalization (documented in brain CTA/MRA at 24 h for EXTEND‐IA and MR CLEAN) or complete reperfusion (documented in brain CT perfusion/MR perfusion at 27 h for SWIFT PRIME) across different RCTs.

**Figure 3:**
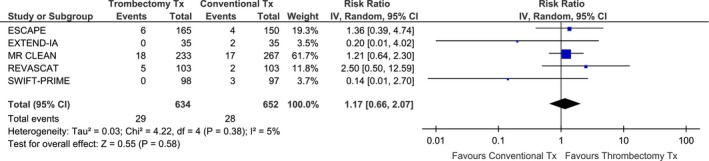
Association of mechanical thrombectomy (vs. best medical therapy) with the likelihood of symptomatic intracranial hemorrhage (sICH) across different RCTs.

**Figure 4:**
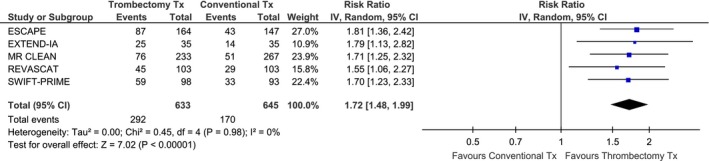
Association of mechanical thrombectomy (vs. best medical therapy) with the likelihood of functional independence at 3 months (modified Rankin scale score of 0–2) across different RCTs.

High complete recanalization rates were documented at the end of the endovascular procedures in all RCTs ranging from 59% in MR CLEAN to 88% in SWIFT PRIME, while follow‐up neuroimaging studies at 24–27 h reported similar rates for persistent recanalization/reperfusion (Table 3). The pooled complete recanalization rate at the end of endovascular procedure was 74% (95% CI: 63%–83%; Fig. 1), but there was evidence of substantial heterogeneity across the five RCTs (*I* ^2^ = 85%, *P* for Cohran *Q* < 0.001). Compared to BMT, MT was associated with an increased probability of complete recanalization/reperfusion at 24–27 h from symptom onset (RR: 2.22; 95% CI: 1.89–2.62; *P* < 0.00001; Fig. 2) and there was no evidence of heterogeneity across trials (*I* ^2^ = 0%). The absolute benefit increase was 44% with MT and that corresponded to a number needed to treat (NNT) of 2. Increased complete recanalization rates resulted in reduced infarction volume at 24–27 h after treatment, a difference that was statistically significant in two of the three trials that provided relative data (Table 4). MT was not associated with an increased risk of sICH (RR: 1.17; 95% CI: 0.66–2.07; *P* = 0.58; Fig. 3) without any evidence of significant heterogeneity across trials (*I* ^2^ = 5%, *P* for Cohran *Q* = 0.38). More specifically, the pooled sICH rates were 4.6% and 4.3% in the MT and BMT groups, respectively. Compared to BMT, MT was associated with an increased likelihood of functional independence (modified Rankin scale score of 0–2) at 3 months (RR: 1.72; 95% CI: 1.48–1.99; *P* < 0.00001; Fig. 4) and there was no evidence of heterogeneity across trials (*I* ^2^ = 0%). The absolute benefit increase was 16% with MT (42% vs. 26% with BMT) and that corresponded to an NNT of 6.

**Table 3:** Complete recanalization rates defined by a modified Thrombolysis in Cerebral Infarction (TICI) score IIb or III at the end of mechanical thrombectomy (second and third column) and by noninvasive neuroimaging 24–27 h later (fourth and fifth column)

Clinical trial	MT	BMT	MT	BMT
MR CLEAN	115/196 (59%)	N/A	141/187 (75%)^a^	68/207 (33%)^a^
ESCAPE	113/156 (72%)	N/A	N/A	N/A
EXTEND‐IA	25/29 (86%)	N/A	33/35 (94%)^a^	15/35 (43%)^a^
SWIFT PRIME	73/83 (88%)	N/A	53/64 (83%)^b^	21/52 (40%)^b^
REVASCAT	67/102 (66%)	N/A	N/A	N/A

MT, mechanical thrombectomy; BMT, best medical therapy; N/A, not applicable.

a Recanalization shown in brain CTA/MRA at 24 h.

b Reperfusion shown in brain CT perfusion/MR perfusion at 27 h.

**Table 4:** Brain infarction volume at 24 h after treatment measured with CT in MR CLEAN trial and with CT or MRI in SWIFT PRIME and REVASCAT trials

Clinical trial	MT (mean, 95% CI)	BMT (mean, 95% CI)	*P*
MR CLEAN	49 mL (22–96)	79 mL (34–125)	<0.01
ESCAPE	N/A	N/A	–
EXTEND‐IA	N/A	N/A	–
SWIFT PRIME	32 mL (0–503)	35 mL (0–407)	0.09
REVASCAT	16 mL (8–59)	39 mL (12–87)	0.02

MT, mechanical thrombectomy; BMT, best medical therapy.

**Figure 5:**
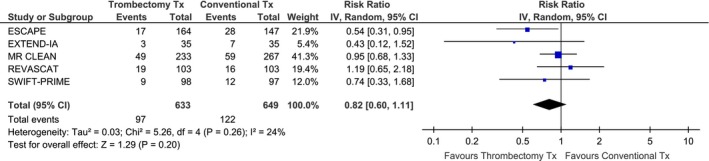
Association of mechanical thrombectomy (vs. best medical therapy) with the likelihood of mortality at 3 months across different RCTs.

Finally, it should be noted that the ESCAPE study was the first phase III RCT evaluating an endovascular therapy that documented a reduction in mortality, but this finding was not reproduced in the other four trials. Consequently, MT was not associated with a decreased risk of 3‐month mortality in the pooled analyses (RR: 0.82; 95% CI: 0.60–1.11; *P* = 0.20; Fig. 5) without any evidence of significant heterogeneity across trials (*I* ^2^ = 24%, *P* for Cohran *Q* = 0.26). The pooled three‐month mortality rates were 15% and 19% in MT and BMT arms, respectively.

## Discussion

Our meta‐analysis of the five recently published endovascular RCTs indicates that MT appears to be a safe and effective treatment for ELVO in the anterior circulation. For every six ELVO patients treated with MT, three more patients will achieve complete recanalization at approximately 24 h following symptom onset and one more will be functionally independent at 3 months in comparison to BMT. Moreover, MT is not associated with an increased risk of sICH or 3‐month mortality and these observations highlight the safety of this specific endovascular approach.

The findings of the five RCTs that were included in our review have also been reproduced in the recent European Stroke Organization conference (Glasgow, April 2015), during which the results of two additional endovascular RCTs were presented. The Assess the Penumbra System in the Treatment of Acute Stroke (THERAPY) trial used the Penumbra aspiration system and enrolled acute stroke patients with large vessel occlusion in the anterior circulation due to clot with a length >8 mm. The trial was prematurely halted after the positive results of the aforementioned trials. There was a trend toward better outcomes and reduced mortality but the prematurely halted study was underpowered to show statistical significance (Mocco et al. ESO Conference 2015). The Trial and Cost Effectiveness Evaluation of Intra‐arterial Thrombectomy in Acute Ischemic Stroke (THRACE) was also prematurely halted and 3‐month intermediate results have been presented (Bracard et al. 2015). Many different catheters were used for AIS patients presenting within 4.5 h if there was no major neurologic improvement (NIHSS decrease more than four points) not only in the anterior but also in the posterior circulation. A statistically significant increase in the primary endpoint (modified Rankin scale score of 0–2 at 3 months) was reported with an absolute benefit increase of 12% (*P* = 0.016) with endovascular therapy.

In the most recent AHA/ASA Focused Update of the 2013 Guidelines for the early management of patients with AIS regarding endovascular treatment, endovascular therapy with a stent retriever is given a Class I, Level of Evidence A recommendation in the setting of acute stroke caused by occlusion of internal carotid artery or proximal middle cerebral artery within 6 h of symptom onset for patients that have already received IVTPA within 4.5 h according to previous guidelines (Powers et al. 2015). IVTPA and thrombectomy are complementary treatment modalities and there has been a report that prior IVTPA may actually act synergistically with thrombectomy by facilitating clot extraction (Guedin et al. 2015). A worldwide reorganization of stroke networks is currently under way in order to provide on time thrombectomy in eligible stroke patients (Lees 2015). Ongoing trials such as the thrombectomy in patients ineligible for iv tPA (THRILL) trial (Bendszus et al. 2015), the Basilar Artery International Cooperation Study (BASICS) (van der Hoeven et al. 2013), and the PerfusiOn Imaging Selection of Ischemic Stroke Patients for EndoVascular ThErapy (POSITIVE) trial (ClinicalTrials.gov NCT01492725) will further clarify potential additional MT indications (posterior circulation, or M2 occlusions) and explore expanded time windows. In addition, a recent large‐scale international collaboration has been announced that will attempt to conduct patient‐level pooled analyses of a large number (>1700) of ELVO patients enrolled in different endovascular trials (Khatri et al. 2015).

The aforementioned findings have important implications for both secondary referral hospitals and developing countries with limited availability of neurointervention services. Severely disabled patients with large proximal arterial occlusions, especially if they were previously very functional, should be immediately transferred from the referral hospital to the nearest stroke center with expertise on endovascular reperfusion therapies (O'Carroll et al. 2015). Therefore, there is a great urgency for the development of organized strokes care systems, especially in developing countries, to identify and directly transport stroke patients with a high suspicion of proximal artery occlusion immediately to comprehensive stroke centers, instead of primary or secondary referral hospitals (Prabhakaran et al. 2015).

In conclusion, endovascular stent thrombectomy is a Level A/Class I evidence‐based treatment for patients with ELVO in anterior circulation. This highly effective and rapid reperfusion therapy substantially improves functional outcomes in patients presenting with the most devastating subtype of AIS. Health systems therefore need to be reorganized to offer this treatment to all appropriate candidates as quickly as possible. Further research is needed to refine patient selection criteria and potentially expand MT indications to a larger number of patients with ELVO.

## Conflict of Interest

None declared.
